# Long-term breeding phenology shift in royal penguins

**DOI:** 10.1002/ece3.281

**Published:** 2012-07

**Authors:** Mark A Hindell, Corey J A Bradshaw, Barry W Brook, Damien A Fordham, Knowles Kerry, Cindy Hull, Clive R McMahon

**Affiliations:** 1 Institute for Marine and Antarctic Studies, University of TasmaniaPrivate Bag 129, Hobart, Tasmania 7001, Australia; 2 The Environment Institute and School of Earth and Environmental Sciences, The University of AdelaideAdelaide, South Australia 5005, Australia; 3 South Australian Research and Development InstituteP.O. Box 120, Henley Beach, South Australia 5022, Australia; 4 Australian Antarctic Division203 Channel Highway, Kingston, Tasmania, 7050, Australia; 5 Department of Zoology, University of TasmaniaPrivate Bag 05, Hobart, Tasmania 7001, Australia; 6 Research Institute for the Environment and Livelihoods, Charles Darwin UniversityDarwin, Northern Territory, 0909, Australia

**Keywords:** Antarctica, egg laying date, global warming, reproduction, seabirds

## Abstract

The Earth's climate is undergoing rapid warming, unprecedented in recent times, which is driving shifts in the distribution and phenology of many plants and animals. Quantifying changes in breeding phenology is important for understanding how populations respond to these changes. While data on shifts in phenology are common for Northern Hemisphere species (especially birds), there is a dearth of evidence from the Southern Hemisphere, and even fewer data available from the marine environment. Surface air temperatures at Macquarie Island have increased by 0.62°C during the 30-year study period (0.21°C decade^−1^) and royal penguins (*Eudyptes schlegeli*) commenced egg laying on average three days earlier in the 1990s than during the 1960s. This contrasts with other studies of Southern Ocean seabirds; five of nine species are now breeding on average 2.1 days later than during the 1950s. Despite the different direction of these trends, they can be explained by a single underlying mechanism: resource availability. There was a negative relationship between the Southern Annular Mode (SAM) and median laying date of royal penguins, such that low-productivity (low SAM) years delayed laying date. This accords with the observations of other seabird species from the Antarctic, where later laying dates were associated with lower sea ice and lower spring productivity. The unifying factor underpinning phenological trends in eastern Antarctica is therefore resource availability; as food becomes scarcer, birds breed later. These changes are not uniform across the region, however, with resource increases in the subantarctic and decreases in eastern Antarctica.

## Introduction

The Earth's biota is beginning to respond to a rapidly changing climate (Parmesan 2006; Traill et al. 2010), and there is evidence that marine systems are particularly sensitive to this change (Richardson and Poloczanska 2008). An early indication of climate-induced changes in an ecosystem is altered phenology of its constituent biota. This refers to changes in the sequence and timing of key events in a species’ annual cycle. Many plants and animals have already demonstrated an advance (i.e., occurring earlier in the year) in phenology over the last century in response to a warming climate, such as the timing of spring flowering, development rate, emergence, first reproduction, and migration (Hughes 2000; Walther et al. 2002; Parmesan and Yohe 2003; Dunn 2004). However, determining the mechanisms driving phenological shifts, such as habitat availability or food resources, is not straightforward because these can be complex and multi-factorial (Parmesan 2006).

Many of the documented changes in phenology are from Northern Hemisphere terrestrial systems, with relatively little evidence from Southern Hemisphere or marine systems (Richardson and Poloczanska 2008). The Southern Ocean in particular has received little attention despite its wide variation in warming trends and large influence on the world's climate (Forcada and Trathan 2009; Mayewski et al. 2009). Unlike the Arctic, permanent human habitation in the Southern Ocean has only occurred in the last 50 years, and even that has been restricted to a small number of scientific bases. This means that the long-term studies needed to detect phenological changes are uncommon (McMahon and Burton 2005; Barbraud and Weimerskirch 2006; Richardson and Poloczanska 2008). Consequently, there are few studies documenting phenological changes in seabirds in this region, in stark contrast to the plethora of studies from the Northern Hemisphere (Miller-Rushing et al. 2008 and papers therein). A meta-analysis by Barbraud and Weimerskirch (2006) found that 44% of nine Antarctic seabird species were breeding later in response to shifts in climate. (The others show no significant change.) This is at odds with observations in the Northern Hemisphere, where arrival and laying date generally occur earlier (Table 1). While it is often difficult to attribute a causal mechanism to phenological changes in the far south, there is some evidence that lowered resource availability arising from changes in winter sea ice extent could be driving the change (Barbraud and Weimerskirch 2001; Rotella et al. 2009).

**Table 1:** Summary of the rates of change (β) in four breeding phenology parameters: laying date—LD, hatching date—HD, ringing date—RD, and date of first eggs—FE, reported in seabirds. Ringing date is a proxy for arrival and laying dates (Frederiksen et al. 2004)

Species	Change rate β (days year^−1^)	Phenology parameter	Span	Latitude	References
Southern Hemisphere					
*Eudyptula minor*	0.040	LD	1968–1998	−38°28′	3
*Eudyptes schlegeli*	−0.108	LD	1964–1999	−54°36′	9
*Aptenodytes forsteri*	n.s.	LD	1950–2005	−66°70′	1
*Pygoscelis adeliae*	0.086	LD	1950–2005	−66°70′	1
*Daption capense*	0.078	LD	1950–2005	−66°70′	1
*Pagodroma nivea*	n.s.	LD	1950–2005	−66°70′	1
*Stercorarius maccormicki*	−0.072	LD	1950–2005	−66°70′	1
*Pygoscelis adeliae*	0.259	HD	1995–2005	−74°20′	7
Mean	0.047				
Northern Hemisphere					
*Rissa tridactyla* (St Paul)	−0.646	HD	1975–2005	57°	2
*Rissa tridactyla* (St George)	−0.578	HD	1975–2005	57°	2
*Rissa brevirostris* (St Paul)	−0.883	HD	1975–2005	57°	2
*Rissa brevirostris* (St George)	−0.792	HD	1975–2005	57°	2
*Uria lomvia* (St Paul)	0.468	HD	1975–2005	57°	2
*Phalacrocorax aristotelis*	−0.230	RD	1969–2002	56°11′	4
*Uria aalge*	0.280	LD	1982–2002	56°11′	4
*Rissa tridactyla*	0.500	FE	1981–2002	56°11′	4
*Ptychoramphus aleuticus*	1.455	HD	1996–2006	50°52′	6
*Fratercula cirrhat*	−0.790	HD	1975–2002	50°35′	5
*Alle alle*	−0.100	HD	1963–2008	77°00′	8
*Rissa tridactyla*	0.000	HD	1963–2008	77°00′	8
Mean	−0.110				

References: 1 = Barbraud and Weimerskirch (2006); 2 = Byrd et al. (2008); 3 = Chambers (2004a, b); 4 = Frederiksen et al. (2004); 5 = Gjerdrum et al. (2003); 6 = Hipfner (2008); 7 = Pezzo et al. (2007); 8 = Moe et al. (2009); 9 = this study.

Given the abundant physical evidence for climate shifts in the Antarctic (Mayewski et al. 2009), it is likely that pheno-logical shifts in subantarctic seabirds foraging in the Southern Ocean will mirror those few studies examining such effects in Antarctic species (McMahon and Burton 2005; Barbraud and Weimerskirch 2006; Richardson and Poloczanska 2008). Seabirds are sensitive indicators of change in marine eco-systems because they integrate the effects of climate forcing on lower trophic levels in ways that are relatively easy to quantify: for example, via changes in breeding times. Within the subantarctic avifauna, macaroni penguins (*Eudyptes chrysolophus*) and their congeners, royal penguins (*E. schlegeli*), are particularly well suited to such an investigation because they are abundant and an important consumer of Southern Ocean euphausids, fish, and squid (Goldsworthy et al. 2001) and they also display highly synchronous breeding. Royal penguins are endemic to Macquarie Island, where there are approximately 850,000 breeding pairs (Copson and Rounsevell 1987). The population has been studied during two periods since the establishment of a scientific base in 1948; over several years in the 1960s and again during the 1990s. The thirty-year gap between these studies offers an invaluable opportunity to quantify phenological changes in a subantarctic seabird and how the species might be responding to environmental changes.

Based on previous work on seabirds, our *a priori* expectation was that there is a relationship between climate and breeding times mediated by food availability (Barbraud and Weimerskirch 2006; Forcada and Trathan 2009). Our aim was therefore to extend the observations of phenological shifts in the Southern Ocean, drawing on a previously untapped historical data resource. We used a three-stage approach to addressing this broader question by (1) establishing the presence any long-term climate trends at Macquarie Island using air temperature records, (2) then assessing the trends in egg laying dates of royal penguins at Macquarie Island, and (3) exploring possible mechanisms that might explain the phenological changes.

## Materials and Methods

### Trends in air temperature at Macquarie Island: 1948–2007

The first step in such a phenological study is to ascertain that there has been a detectable change in a climate variable over the course of the study. In the absence of long-term sea surface temperature records from the Southern Ocean, which are only available since the late 1970s, we assessed the regional trends in climate using daily maximum ground-level air temperatures collected at Macquarie Island since the establishment of the scientific base there in 1948 (http://www.bom.gov.au/climate). Air temperatures, particularly on small oceanic islands, are closely related to surrounding ocean temperatures (Mayewski et al. 2009). It should be noted however, that although air temperature and sea temperature are related, there is no clear mechanistic link between air temperature and timing of breeding. The data were expressed as average annual values. While using an annual average can obscure underlying seasonal trends, it is sufficient to detect long-term trends.

### Trends in laying date

**Figure 1:**
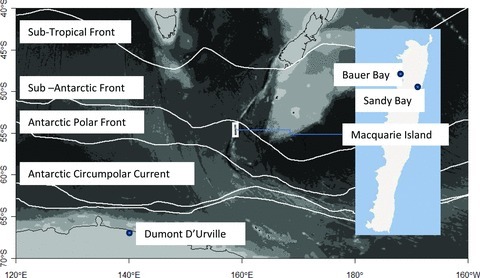
Map of the East Antarctic Southern Ocean showing bathymetry and the major oceanographic features. Inset: Macquarie Island, indicating the location of the colonies examined (Bauer Bay [1960s] and Sandy Bay [1990s]).

Information on median egg laying date was available from Macquarie Island based on two separate studies in the 1960s and the 1990s. The 1960s data were collected as part of a larger banding study investigating the demographics of royal penguins (Carrick 1972), and involved daily checks of individually flipper-banded nesting penguins within the Bauer Bay colony (Fig. 1). The study was conducted from 1955 to 1970, but there were only sufficient data to quantify laying dates in seven of those years (1964–1969). The date of egg laying for each pair was taken as the first day in which an egg was reported as present. The data from the 1990s were collected as part of a study of the breeding biology of royal penguins at the Sandy Bay colony on the east coast of the island (Hull and Wilson 1996). This study went from 1993 to 1997, with 50 nests monitored daily in each year. The nests were distributed along three transects equally spaced along the length of the colony. All birds were flipper-banded after pair formation. Again, the date of egg laying for each pair was taken as the first day in which an egg was reported present. We calculated median date of egg laying of the first egg (the *A* egg; royal penguins lay two eggs and always reject the first [Carrick 1972] for each year from the records of individual nests.

### Mechanisms that may explain the phenological changes

At other islands, the closely related macaroni penguin's foraging is concentrated around oceanic frontal zones and determined by the distribution of prey within those zones (Thiebot et al. 2011). This is, in turn, determined by the biophysical characteristics of the water column (Sokolov and Rintoul 2002), themselves being influenced by climatic events (Carleton and Carpenter 1990; Ledley and Huang 1997; Turner 2004). There are no direct measures of the distribution and abundance of royal penguin prey species over the time scales required for this study. Rather, we used the Southern Annular Mode (SAM) as a broad-scale proxy for Southern Ocean productivity. The SAM is a large-scale alteration of atmospheric mass between the mid and high latitudes (Baldwin and Thompson 2009), and is characterized by pressure anomalies of one sign centered in the Antarctic (∼65°S) and anomalies of the opposite sign centered over about 40°S. We use the SAM index calculated from sea-level pressure (SLP) anomalies south of 20°S (http://jisao.washington.edu/data/aao/slp/#analyses). The SAM is a reasonable proxy for large-scale biological productivity, with higher productivity in years of higher SAM index (Shinsuke et al. 2003; Lefebvre et al. 2004; Ainley and Blight 2008; Yuan and Li 2008; Forcada and Trathan 2009). Variability in large-scale climate indices (e.g., El Niño Southern Oscillation) have been linked to variation in foraging performances in other predators, such as elephant seals (*Mirounga leonina*), king penguins (*Aptenodytes patagonicus*), and blue petrels (*Halobaena caerulea*) (Guinet et al. 1998; Bradshaw et al. 2004; Cotte et al. 2007).

We assessed the relationship between SAM and median laying date by comparing a general linear model (GLM) of laying date∼sea level *SAM* to a null (intercept only) model GLM (laying date∼1). The comparison was based on the ratio of weighted Bayes Information Criteria (*w*BIC*_c_*) of the two models.

## Results

### Trends in air temperature: 1948–2007

**Figure 2:**
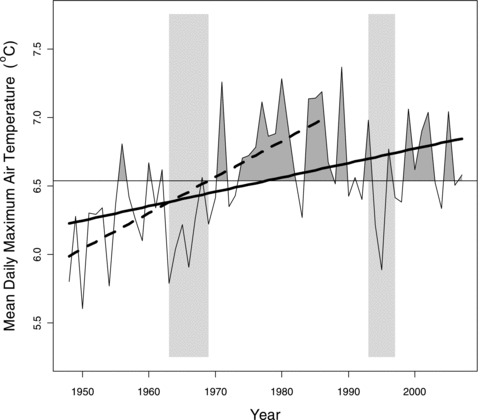
Mean daily maximum air temperature recorded each year between 1948 and 2007 at Macquarie Island. Data expressed relative to the overall mean over that period (6.54°C), with periods above that value shaded and those below in white. Also indicated is the line of best fit from the regression of year against the annual mean. The dotted line is the trend calculated for the period 1948–1986 (slope = 0.026, *R*^2^ = 0.46) (Adamson et al. 1988). Shaded rectangles represent periods during which laying data were collected.

Air-temperature data were collected for 60 years at Macquarie Island, from 1948 to 2007 (Fig. 2). The data were collected at the meteorological station on the Isthmus, approximately 10 km north of the penguin colonies used in this study. During that time, there was an increase in the annual mean daily maximum, so that a GLM describing a change in temperature over time (% deviance explained = 20.2) was 998 times more likely than the intercept-only GLM describing no change in temperature over time (based on the ratio of weights, i.e., *w*BIC*_c_* annual mean maximum temperature∼year/wBIC_c_ Null). Temperature increased at an average rate of 0.019 ± 0.003°C year^−1^: from 6.22°C (modeled values) in 1948 to 6.85°C in 2007 (mean total increase of 0.62°C). The mean annual temperature during the first six-year phase of the study was 6.1°C compared to 6.5°C in the second phase between 1993 and 1997 (Fig. 2).

### Laying dates

**Figure 3:**
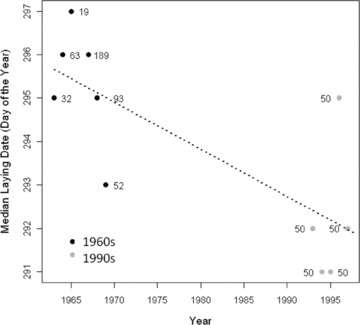
Median laying dates (day of the year) for each of the 11 years for which data were available. The numbers adjacent to each point are the number of nests monitored in that year. Dotted line is line of best fit from a least-squares regression of laying date against year (laying date = 508.51 − 0.108 × year).

On average, 75 nests were monitored each year during the 1960s, compared to 50 during the 1990s. The median laying dates in the 1960s ranged from 21 to 25 October (overall median = 23 October), while those from the 1990s ranged from 19 to 22 October (overall median = 20 October). This represents a decrease in laying date over 34 years of 3.5 days (Fig. 3). A GLM describing the change in laying date between the 1960s and the 1990s (% deviance explained = 57.1) was 14.7 times more likely than the intercept-only GLM describing no change in laying date. The mean rate of change in egg laying date over 34 years was –0.108 days year^−1^ (i.e., a shift of 10.8 days earlier per century; Fig. 2).

### Oceanic conditions and laying date

**Figure 4:**
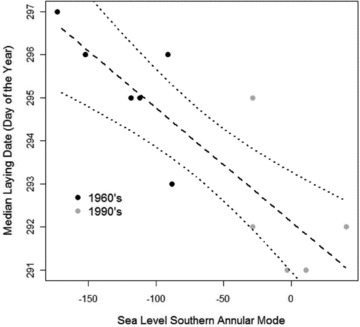
The relationship between median laying dates (day of the year) and the Sea Level Southern Annular Mode (SAM) of that year. The central line represents the predicted laying date, and the dashed lines are the 95% confidence interval of that prediction.

Median laying date was strongly influenced by the SAM. The GLM relating laying date to SAM (% deviance explained = 72.7) was 332.3 times more likely than the intercept-only GLM describing no relationship with SAM (*w*BIC*_c_* median laying date∼SAM = 0.997, *w*BIC*_c_* Null = 0.003, delta BIC = 11.89). This relationship was negative, with a slope of –0.026 (SE = 0.0054) days per unit of SAM (Fig. 4).

## Discussion

We established that there was a long-term climate trend at Macquarie Island. The maximum air temperature over the 40 years increased by 0.62°C, although the mean annual rate (0.01°C year^−1^) was lower than the 0.02°C year^−1^ increase reported globally (Hansen et al. 2006). Our calculated temperature trends at Macquarie Island are also lower than those reported for Macquarie between 1949–1986 (0.026°C year^−1^; Fig. 2) by Adamson et al. 1988. This is due to the longer time series used in our study that incorporated several years of relatively cool temperatures in the 2000s. Rates of warming across the globe vary considerably both spatially and temporally (Hansen et al. 2006; Monaghan and Bromwich 2008), with even similar-latitude subantarctic islands showing different rates (e.g., Marion Island is warming at a mean of 0.028°C year^−1^) (le Roux and McGeoch 2008).

Concurrent with this, there was a clear advance in the median laying date of royal penguins at Macquarie Island, so that in the 1990s penguins were laying on average 3.5 days earlier than in the 1960s. Of the eight Southern Ocean seabirds for which phenological trends have been investigated (including our study), four now breed later than they did during the 1950s and 1960s (Table 1). Three penguin species (Adelie, Gentoo, Chinstrap) bred earlier on the Antarctic Peninsula (Lynch et al. 2009), a region with very different patterns of climate change (Vaughan et al. 2003). The trend in eastern Antarctica is in contrast to studies of seabirds in the Northern Hemisphere where the nature of the phenological shift is more variable, both in terms of direction (earlier *versus* later breeding) and the rate of change. How a species responds to climate change depends on the complex interplay between its life-history characteristics, its habitat requirements, and the physical environment (Forcada and Trathan 2009), so it is not surprising that species respond and potentially adapt to warming trends differently across the globe.

Our study used two different breeding colonies on opposite sides of the island, and this may be a confounding factor when interpreting the changes in laying dates. There are both on-shore and off-shore factors that could influence the phenology at different colonies. With respect to on-shore factors, we argue that timing of breeding will be proximally caused by their date of arrival back on the island, not the local conditions that they find when they get there. For example, although the west coast is more exposed, it is difficult to think of a mechanism by which this will influence the laying date. It will always be more exposed, so the birds will have to deal with those local conditions at some time. With respect to off-shore factors, the penguins are likely to be foraging south of the island in association with the Antarctic Polar Front during the winter prelaying period, based on the foraging behavior of the congeneric Macaroni penguins at other islands (Barlow and Croxall 2002; Bost et al. 2009). Birds from both colonies will therefore have similar distances to travel between their foraging and breeding areas. Further, intra island comparisons of Macaroni penguins at Heard Island have found that birds from colonies at opposite ends of the island (also with different local conditions) use similar foraging grounds (Hindell et al. 2011). This is quite a different situation to other species, such as Adelie Penguins, where intra island differences in laying dates are due to the need for birds to walk over sea-ice to reach their colonies that are different distances from the ice edge (Ballard et al. 2010). It is therefore unlikely that the location of the colonies in our study influences arrival and laying dates. Finally, our finding of a correlation between the SAM and timing of breeding suggests that the drivers for arrival at the colony are large spatial scale factors. In this context, it is immaterial what side of Macquarie Island the penguins are breeding on.

Royal penguins bred earlier in years when the SAM was relatively high, which equates to higher productivity and more abundant food in the Southern Ocean at that time (Sarmiento et al. 2004). Penguins need to attain a threshold body condition before being able to produce eggs (Norman et al. 1992), so egg laying date is probably tightly linked to foraging success beforehand. In general, food abundance and quality drive the timing of bird reproduction (Both and Visser 2005), but interpreting these observations is not straightforward because changes in breeding dates can have different underlying causes (Carey 2009). For example, little auks (*Alle alle*) in Svalbard breed earlier in response to increasing air temperatures (Moe et al. 2009). While this is the same general pattern we found for royal penguins, auks breed earlier due to early snow melt (clearing) in their breeding colonies and not because of climate-driven variation in food availability.

The SAM has been in a long-term positive phase since at least the 1970s (Marshall 2003), making it difficult to dis-entangle causation from correlation for the change in breeding times of royal penguins. However, phenological changes in other high-Antarctic species have also been related changing food availability (Barbraud and Weimerskirch 2006), in those cases mediated by the extent of winter sea ice (Loeb et al. 1997; Nicol et al. 2000). Clutch initiation date of Adelie penguins is also negatively correlated with SAM (Emmerson and Southwell 2011). The fact that these other studies have linked changes in phenology to food availability supports our finding that changes in royal penguin breeding timetables are linked to the birds’ resource base.

There is now considerable evidence that seabird breeding behavior and performance (e.g., Frederiksen et al. 2004; Gaston et al. 2005; Durant et al. 2006) is driven predominantly by food availability, such that resource-plenty years tend to result in earlier laying and hatching (Suddaby and Ratcliffe 1997; Abraham and Sydeman 2004; Møller et al. 2006; Ramos et al. 2006; Monticelli et al. 2007). There is also some evidence from lower latitude regions that years with warmer sea-surface temperatures (equating to higher resource availability) correspond to an early start of the breeding season in little penguins (*Eudyptula minor*) (Chambers 2004b; Cullen et al. 2009).

The demographic consequences of a 3.5-day change in laying dates are unclear. In many Northern Hemisphere systems, changing arrival dates are desynchronizing the peaks in chick production and prey availability (Dickey et al. 2008; Thackeray et al. 2010), and this has the potential to reduce juvenile survival and population growth rate. In the Southern Ocean, there is as yet no evidence for this type of mismatch. However, in the case of reduced food availability leading to later laying, it is likely that resource-poor years will be further reflected in either longer periods of chick care and or reduced breeding success, which would have demographic consequences such as altering age structure and reducing population growth rate (Møller et al. 2006; Lescroel et al. 2009).

The long-term datasets needed to investigate phenology changes in long-lived and wide-ranging species are rare, and even rarer in the Antarctic due to the lack of permanent human presence and remoteness. To assess the biological consequences of climate change, it is not sufficient to demonstrate phenological changes in relation to a shift in a climatic parameter such as temperature. Our study provides new insights into how changes in the environment affect seabirds in the Southern Ocean and demonstrates that in the Southern Ocean, disparate regional patterns in phenology (i.e., later and earlier laying dates) arise from the same proximate cause—food availability.
